# ANMDA: anti-noise based computational model for predicting potential miRNA-disease associations

**DOI:** 10.1186/s12859-021-04266-6

**Published:** 2021-07-02

**Authors:** Xue-Jun Chen, Xin-Yun Hua, Zhen-Ran Jiang

**Affiliations:** School of Computer Science and Technology, East China Normal University, Shanghai, 200062 China

**Keywords:** miRNA-disease association, *k*-means, Noise smoothing, Light gradient boosting machine

## Abstract

**Background:**

A growing proportion of research has proved that microRNAs (miRNAs) can regulate the function of target genes and have close relations with various diseases. Developing computational methods to exploit more potential miRNA-disease associations can provide clues for further functional research.

**Results:**

Inspired by the work of predecessors, we discover that the noise hiding in the data can affect the prediction performance and then propose an anti-noise algorithm (ANMDA) to predict potential miRNA-disease associations. Firstly, we calculate the similarity in miRNAs and diseases to construct features and obtain positive samples according to the Human MicroRNA Disease Database version 2.0 (HMDD v2.0). Then, we apply *k*-means on the undetected miRNA-disease associations and sample the negative examples equally from the k-cluster. Further, we construct several data subsets through sampling with replacement to feed on the light gradient boosting machine (LightGBM) method. Finally, the voting method is applied to predict potential miRNA-disease relationships. As a result, ANMDA can achieve an area under the receiver operating characteristic curve (AUROC) of 0.9373 ± 0.0005 in five-fold cross-validation, which is superior to several published methods. In addition, we analyze the predicted miRNA-disease associations with high probability and compare them with the data in HMDD v3.0 in the case study. The results show ANMDA is a novel and practical algorithm that can be used to infer potential miRNA-disease associations.

**Conclusion:**

The results indicate the noise hiding in the data has an obvious impact on predicting potential miRNA-disease associations. We believe ANMDA can achieve better results from this task with more methods used in dealing with the data noise.

**Supplementary Information:**

The online version contains supplementary material available at 10.1186/s12859-021-04266-6.

## Background

MicroRNA (miRNA) is a class of endogenous small molecule single-stranded non-coding RNA (ncRNA), which can specifically bind to 3'-UTR (3'-untranslated region) of the target mRNA [1]. Research shows that miRNA is involved in many cell activities including cell proliferation, apoptosis, and stem cell differentiation [2, 3]. It's reported that 48,860 different mature miRNAs sequences have been found from 271 organic organisms, of which 2654 mature miRNAs sequences come from humans [4].

MiRNA-related malfunctions are related to various types of human diseases including tumor, neurodegeneration, and diabetic cardiomyopathy, etc. [5–7]. Therefore, uncovering the miRNA-disease associations can provide valuable clues for disease diagnosis at an early stage [8]. Based on the hypothesis that miRNAs with similar functions tend to be related to similar diseases [9], much effort has been devoted to developing various computational methods for miRNA-disease associations prediction during the past years [10].

In general, there are four main types of methods proposed to predict potential miRNA-disease associations.

One type of method is the score function-based algorithms. Jiang et al. [11] integrated miRNAs functional interactions network and disease similarity network and then implemented a scoring method to predict the associations. Chen et al. [12] used a model of calculating within-scores and between scores for miRNA-disease association probabilities (WBSMDA) by integrating miRNA functional similarity, disease semantic similarity, and using Gaussian kernel functions. One challenge of these methods is to utilize more effective features and to design a reasonable score function.

Another type of method is network-based algorithms. Shi et al. [13] tried to connect miRNA and disease through the gene function network and applied the random walk algorithm for final prediction. You et al. [14] constructed a heterogeneous graph with many paths by using weighted matrices to design a path-based algorithm for prediction (PBMDA). Qu et al. [15] built a reliable heterogeneous network and used KATZ to predict miRNA-disease associations (KATZMDA). One challenge of the methods is to integrate different data to build reliable networks and analyze the network function.

The third type of method is mainly based on machine learning algorithms. Chen et al. [16] proposed a ranking-based *k*-nearest neighbor method for miRNA-disease associations prediction (RKNNMDA). RKNNMDA searched miRNA and disease by *k*-nearest neighbors and re-ranked them by support vector machine (SVM). Ha et al. [17] utilized a matrix factorization method to predict miRNA-disease associations (PMAMCA). Zhu et al. [18] used the biased heat conduction (BHCMDA) to pay more attention to unpopular nodes and improve the final results. Recently, ensemble learning methods have been designed to solve this problem and achieve great success. For instance, Zhao et al. [19] adopted the adaptive boosting algorithm for prediction (ABMDA). By adapting the weighing coefficient of residual samples, the algorithm re-learned the residual samples and obtain better results. Zhou et al. [20] combined gradient boosting decision trees with logistic regression (GBDT-LR) to predict potential pairs. Yao et al. [21] used the random forest to select 100 important features and predict miRNA-disease associations based on the selected features (IRFMDA-100). Peng et al. [22] attempted to solve this association inference based on ensemble learning and kernel ridge regression (EKRRMDA). However, the training cost of the ensemble learning methods is often high.

The last type of method belongs to deep learning-based methods. As convolution neural networks (CNN) can obtain potential information between features effectively, Peng et al. [23] used auto-encoders for dimensionality reduction and then applied CNN to predict miRNA-disease associations (MDACNN). To extract dense and high-dimensional representations of diseases and miRNAs, Ji et al. [24] used a deep autoencoder framework (AEMDA). Further, to utilize the information of all miRNA-disease pairs during the pre-training process, Chen et al. [25] adopted a deep-belief network (DBNMDA) to predict the associations. Li et al. [26] applied fully connected graph convolutional networks to rank the potential pairs, which combined the graph-related techniques and CNN (FCGCNMDA). However, deep learning may be more suitable for bigger data.

Although much progress has been made in this field, the noise hiding in the data is an unprecedented problem to be tackled. As some researchers [19–21, 23, 25, 26] regard undetected miRNA-disease pairs as negative samples and randomly choose several samples to feed into algorithms, the algorithms may be influenced by some unreliable negative samples.

This paper proposes a novel anti-noise algorithm predict potential miRNA-disease associations (ANMDA). According to the method, we first analyze the interference of the noise and then use a *k*-means algorithm to pick negative samples, subsample to noise smoothing, and finally apply Light Gradient Boosting Machine (LightGBM) to tackle this problem.

The main contributions are listed as follows: (1) We focus on the noise hiding in the data from a new perspective. (2) We subsample the data to smooth the noise to eliminate the influence of the noise. (3) We apply an effective algorithm (LightGBM) to further deal with the noise. The results demonstrate that ANMDA can outperform some published methods.

## Result

### Experiment design

To validate the performance of ANMDA, we design different experiments to demonstrate the effect of subsampling for noise smoothing and the superiority of LightGBM. In our study, all of the experiments are implemented by using five-fold cross-validation 100 times, and the evaluation metrics are the same as other works including the area under the receiver operating characteristic curve (AUROC), area under the precise-recall curve (AUPR), precision, recall, and F1-score.

### Performance evaluation on ANMDA

We evaluate the performance of ANMDA and compare the results of ANMDA with 6 other published methods: WBSMDA, BHCMDA, EKRRMDA, MDACNN, FCGCNMDA, and DBNMDA. The main character for each method is shown in Table 1. WBSMDA is a classic method, BHCMDA and EKRRMDA are recently published machine learning methods, EKRRMDA is an ensemble learning method and more comparable to ANMDA. Furthermore, the deep learning-based models: MDACNN, FCGCNMDA, and DBNMDA are also picked.

**Table 1:** The main ideas of ANMDA and 6 published methods

Method	Main idea
ANMDA	Adopts subsampling for noise smoothing and light gradient boosting machine for prediction
BHCMDA	Uses biased heat conduction-based method to pay attention to specific nodes for prediction
DBNMDA	Constructs deep-belief network for prediction
EKRRMDA	Applies ensemble learning and kernel ridge regression on various data subset created by random selection of features for prediction
FCGCNMDA	Applies fully connected graph convolutional networks for prediction
MDACNN	Uses auto-encoders for dimensionality reduction and then applies convolutional neural networks for prediction
WBSMDA	Calculates within-scores and between scores for prediction

**Fig. 1:**
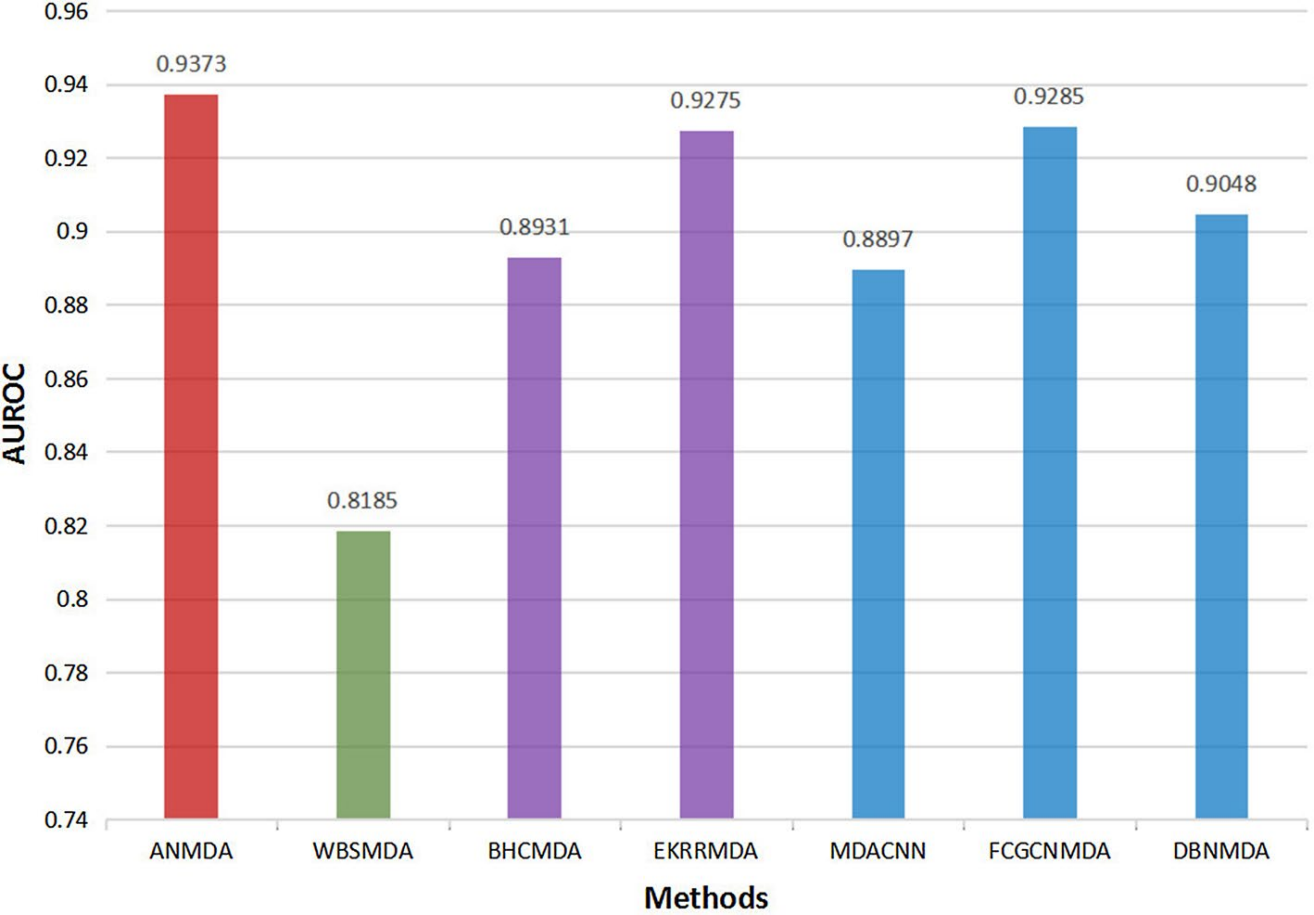
The AUROCs of ANMDA and other 6 published methods

The AUROCs of ANMDA and other 6 published methods are shown in Fig. 1, as we can see, ABMDA achieves the best performance in these 6 methods. What’s more, the standard deviation of ANMDA is 0.0005, which means that ANMDA is more stable than other methods such as WBSMDA (0.0009) and DBNMDA (0.0026).

**Fig. 2:**
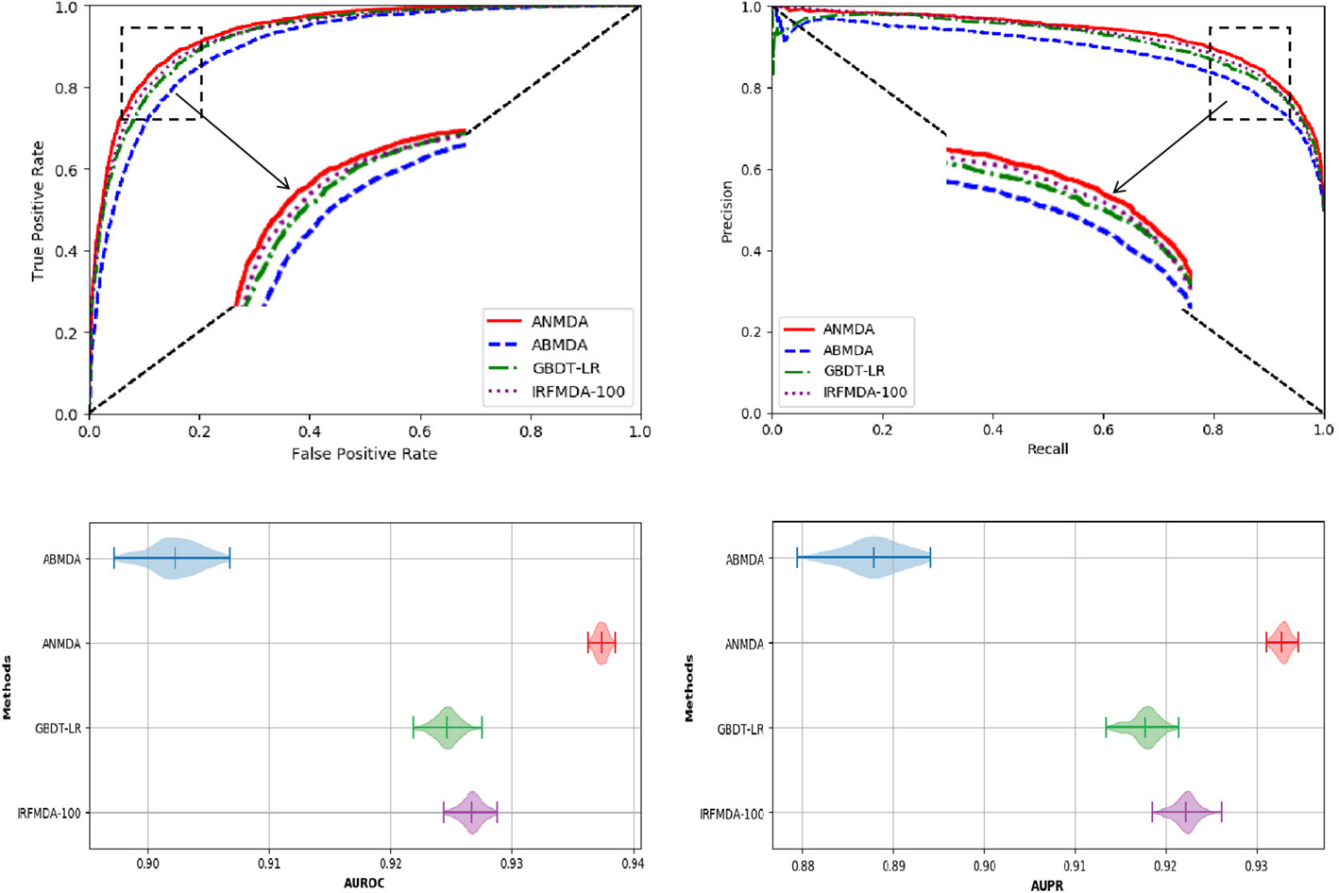
The performance of ANMDA, ABMDA, GBDT-LR and IRFMDA-100 tested on the same data

To further show the performance of ANMDA, we repeat ABMDA, GBDT-LR, and IRFMDA-100 to compare with ANMDA because they have similar feature construction and data construction. In addition, all of them belong to ensemble learning algorithms. To design a fair and convincing experiment, we test these methods on the same data. The results are shown in Fig. 2. It is shown from the ROC curve and the precise-recall curve that ANMDA can outperform ABMDA, GBDT-LR, and IRFMDA-100. In addition, ANMDA can achieve higher AUROC and AUPR and lower standard deviation than ABMDA, GBDT-LR, and IRFMDA-100. Table 2 shows the performance of different methods in 100 times five-fold cross-validation test.

**Table 2:** The performance of ANMDA, ABMDA, GBDT-LR and IRFMDA-100 in 100 times five-fold cross validation

Metrics	ANMDA	ABMDA	GBDT-LR	IRFMDA-100
AUROC	0.9373 ± 0.0005	0.9023 ± 0.0021	0.9246 ± 0.0010	0.9267 ± 0.0009
AUPR	0.9328 ± 0.0008	0.8879 ± 0.0032	0.9177 ± 0.0015	0.9222 ± 0.0012
Precision	0.8561 ± 0.0017	0.8213 ± 0.0033	0.8403 ± 0.0026	0.8447 ± 0.0021
Recall	0.8728 ± 0.0020	0.8371 ± 0.0044	0.8567 ± 0.0031	0.8598 ± 0.0025
F1-score	0.8643 ± 0.0014	0.8290 ± 0.0030	0.8484 ± 0.0021	0.8521 ± 0.0016

### Effect of subsampling for noise smoothing

**Fig. 3:**
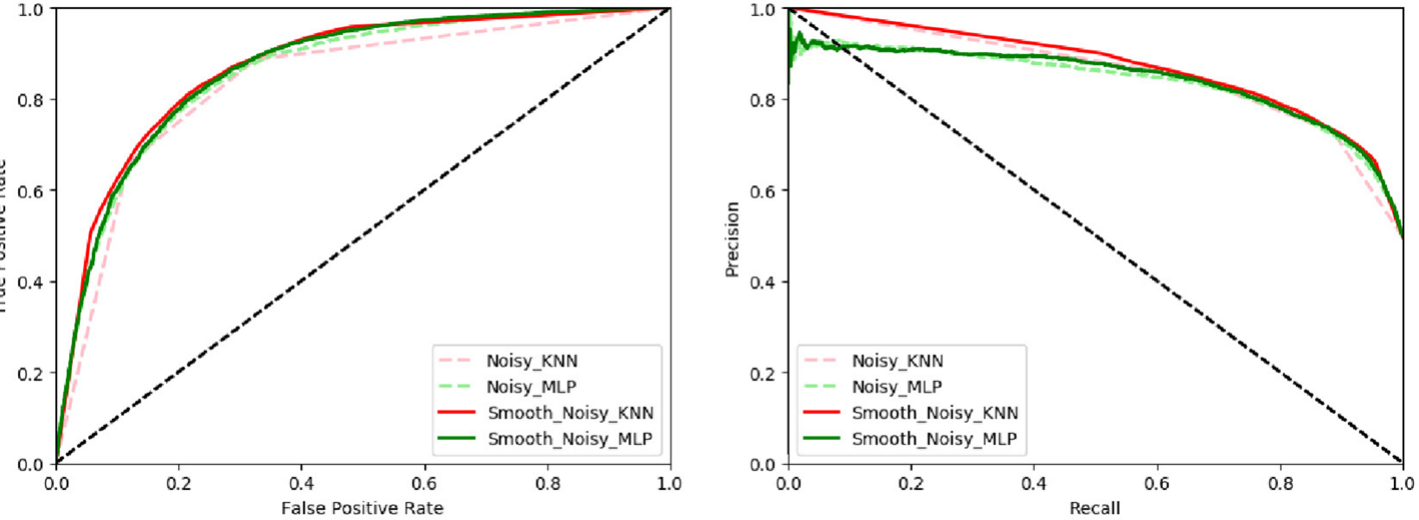
The ROC and PR curves of different algorithms with and without subsampling for noise smoothing

To evaluate the influence of subsampling for noise smoothing, we compare the results of using subsampling for noise smoothing or not. The results are shown in Fig. 3.

Noisy_KNN and Noisy_MLP represent applying *k*-Nearest Neighbor (kNN) and Multilayer Perceptron (MLP) directly for the data, respectively. Smooth_Noisy_KNN and Smooth_Noisy_MLP represent applying kNN and MLP in subsampling for noise smoothing on the data, respectively.

The results demonstrate that the performance of both algorithms is improved after using subsampling for noise smoothing. Specifically, the average AUROC of kNN and MLP increases by 2.35%, and the average AUPR increases by 3.75%, respectively.

### The superiority of LightGBM in noise resistance

**Fig. 4:**
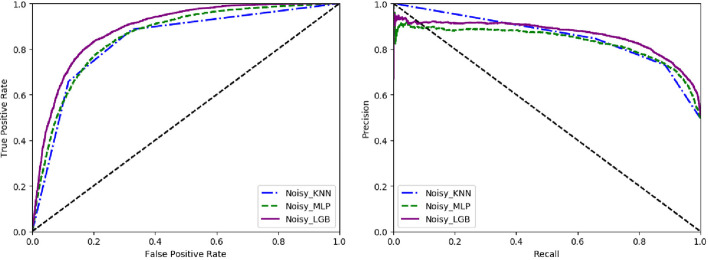
The ROC and PR curves of kNN MLP and LightGBM without subsampling for noise smoothing

To reveal the noise resistance ability of each algorithm, we compare the performance of the methods (LightGBM, kNN, and MLP) on the dataset. The results are shown in Fig. 4.

Noisy_KNN, Noisy_MLP, Noisy_LGB represent applying kNN, MLP LightGBM method, respectively. It can be seen that the performance of LightGBM is better than the other two algorithms, reflecting that LightGBM is expert in dealing with the noise in the data.

### Case study

Further, we use ANMDA to predict undetected miRNA-disease pairs that are not recorded in the Human MicroRNA Disease Database version 2.0 (HMDD v2.0). Then, we verify the results in HMDD v3.0 which records more newly-discovered miRNA-disease associations. The results of the top 200 miRNA-disease associations predicted by ANMDA are shown in the Additional file 1.

Two kinds of case studies are carried out to prove the prediction ability of ANMDA. In the first part, we sort all of the undetected pairs and then verify the top 50 associations predicted by ANMDA with HMDD v3.0. The results are shown in the Additional file 2: Table 1. In the second part, we apply ANMDA to predict prostate neoplasm, gastric neoplasm, colorectal carcinoma, melanoma, and hepatocellular carcinoma. For each disease, the top 10 predicted miRNA-disease associations are selected based on the probabilities. The results are shown in the Additional File 2: Table 2.

In conclusion, the case studies indicate that ANMDA can predict potential miRNA-disease associations with high accuracy.

## Discussion

In this work, we analyze the noise hiding in the data systematically and propose a novel and practical algorithm ANMDA to tackle the noise properly. The main reasons can be listed as follows: (1) By subsampling for noise smoothing, we extract several subsets from the data. In this way, the noise can be separated into each subset, thereby it reduces the interference to the algorithm on judging positive samples because of the noise aggregation. Further, subsampling for noise smoothing can further decrease the influence of the noise by averaging the prediction results of each subset. (2) The residual is mainly caused by the noise hiding in the data. Further, LightGBM based on GBDT can fit residual in each iteration and improve the final prediction.

However, there are also some limitations in ANMDA. First, the high computational cost in the training process of ANMDA is an important problem. For instance, it takes about 300 min to finish five-fold cross-validation 100 times with CPU of Intel Xeon E3-1231 and 1.5 GB of memory usage. In addition, using the current sampling method to discover reliable negative samples is common, therefore, there is still room for improvement.

## Conclusion

This paper proposes a novel method (ANMDA) to predict potential miRNA-disease associations. The experiment results confirm that ANMDA can achieve better results than other published methods. In the case study, several miRNA-disease associations predicted by ANMDA are supported by HMDD v3.0. Therefore, ANMDA is effective and can provide a reference for researchers. In the follow-up work, we plan to use feature selection to accelerate the training process and try to find reliable negative samples. Further, some biological experiments can also be conducted to verify the prediction results of ANMDA.

## Methods

**Fig. 5:**
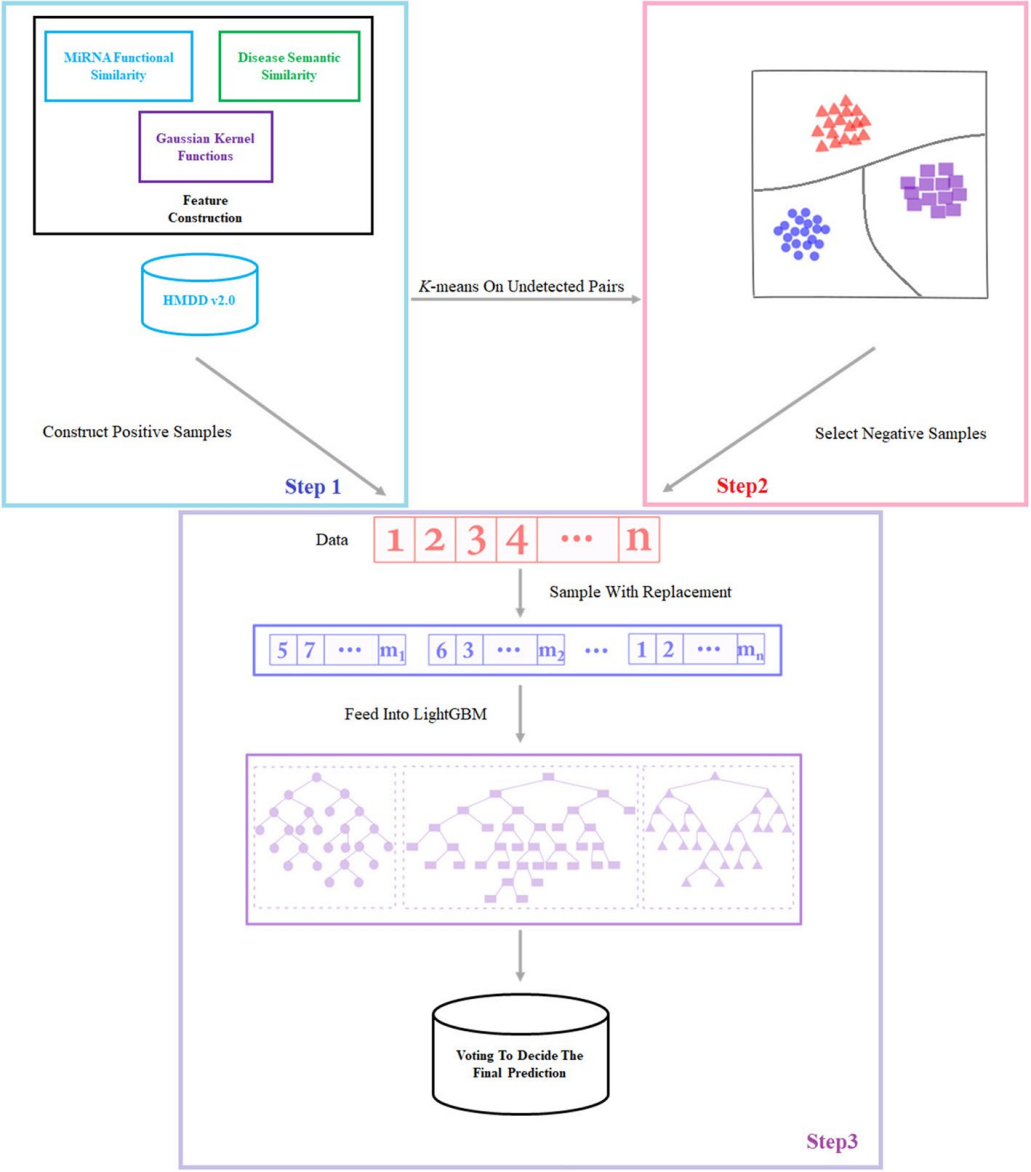
The framework of ANMDA contains three steps: construct features; construct data (construct positive samples and using k-means on undetected pairs to select negative samples based on HMDD v2.0); apply the algorithm to predict the associations

The framework of ANMDA is shown in Fig. 5.

First, the features are constructed based on the miRNA functional similarity, disease semantic similarity, and Gaussian kernel functions. Second, we try to visualize the noise to reveal the effect of noise on data. Based on HMDD v2.0, we construct positive samples and use *k*-means on undetected pairs to select negative samples as data. Then, we subsample the data to smooth the noise. Finally, each subset is fed to LightGBM, and a voting rule is used to decide the final prediction.

### MiRNA-disease associations

HMDD records experimentally supported human miRNA and disease associations. The current version of HMDD is 3.0. As most of the researchers [12–22, 25, 26] choose HMDD v2.0 to test their methods, so we also take it to validate ANMDA. Finally, we obtained 5430 experimentally verified associations, including 495 miRNAs and 383 diseases [27].

### Feature construction

We construct the features by integrating miRNA functional similarity, disease semantic similarity, and using Gaussian kernel functions, which is similar to several other methods [14, 16, 18–22, 24–26].

### Disease semantic similarity

Based on the idea that "functionally similar miRNAs may be associated with similar diseases, vice versa" [28], we calculate the semantic similarity of two diseases according to the extent that they share in common [29].

First, according to MeSH (Medical Subject Headings) tree structure, the relationship between diseases can be displayed as a layered directed acyclic graph (DAG). Each vertex is composed of tree numbers and the heading of one disease. The directed edge in DAG represents the coordination of different diseases. The diseases with a more general heading (like neoplasm) are at an upper layer in the DAG called ancestor nodes. The vertex at a lower layer in the DAG called the children node is composed of diseases having a more specific definition. Given a disease *d*_*i*_ and its DAG Equation is as follows:

1 $$DAG\left( {d_{i} } \right) = \left( {d_{i} ,P(d_{i} ),\;S(d_{i} )} \right)$$

where *P(d*_*i*_*)* represents the set of vertexes in the DAG and *S(d*_*i*_*)* represents the set of edges in the DAG.

Therefore, the similarity based on the semantic value between two diseases can be measured according to their positions in the DAG. The more information two diseases share in common, the more similar they are. To be specific, the semantic similarity between disease *d*_*i*_ and disease *d*_*j*_ can be calculated as follows:

2 $$SS\left( {d_{i} ,d_{j} } \right) = \frac{{\sum\nolimits_{{d \in P\left( {d_{i} } \right) \cap P(d_{j} )}} {\left( {D_{{d_{i} }} (d) + D_{{d_{j} }} (d)} \right)} }}{{V(d_{i} ) + V(d_{j} )}}$$

Respectively, *D*_*di*_*(d)* is defined as the semantic value of the disease *d* contributes to the disease *d*_*i*_. Disease *d* is a set of the vertex shared by the disease *d*_*i*_ and the disease *d*_*j*_ in common in the DAG. *V(d*_*i*_*)* represents the semantic value of the disease *d*_*i*_.

To calculate *D*_*di*_*(d)*, we assume that diseases at different layers in the DAG contribute differently to the semantic value of disease *d*_*i*_ [38]. Therefore, we define it as a semantic contribution factor and the contribution of disease to *d*_*i*_ itself is defined as 1, and the disease located at the upper node of the DAG denotes less to the semantic value of the disease *d*_*i*_. Therefore, the contribution of disease *d* to the semantic value of disease *d*_*i*_ can be calculated by the formula:

3 $$D_{{d_{i} }} (d) = \left\{ {\begin{array}{*{20}c} {1,d = d_{i} } \\ {\max \left( {\Delta \times D_{{d_{i} }} (d^{\prime } )/d^{\prime } \in \;children\;space\;of\;space\;d/} \right),\;d \ne d_{i} } \\ \end{array} } \right.$$

In addition, to avoid the problem that two kinds of diseases having different occurrences in the DAG are calculated as the same semantic value for being at the same layer, a new way is used to define the contribution of disease *d* to the semantic value of disease *d*_*i*_:

4 $$D_{{d_{i} }} (d) = - \log \frac{{N_{d} }}{N}$$

In the formula, *N*_*d*_ is the number of DAGs that contain diseases *d*. *N* represents the number of all of the diseases. Based on the contribution of each disease *d* in the DAG to the disease *d*_*i*_, disease *d*_*i*_’s *V(d*_*i*_*)* can be calculated by the formula:

5 $$V(d_{i} ) = \sum\limits_{{d \in P(d_{i} )}} {D_{{d_{i} }} (d)}$$

As shown in Eqs. (3) and (4), there are two ways to calculate *D*_*di*_*(d)*. Thus, two semantic similarities (*SS*_*1*_ and *SS*_*2*_) are calculated according to Eq. (2). Here, the final semantic similarity is calculated as follows:

6 $$SS\left( {d_{i} ,d_{j} } \right) = \frac{{SS_{1} \left( {d_{i} ,d_{j} } \right) + SS_{2} \left( {d_{i} ,d_{j} } \right)}}{2}$$

### miRNA functional similarity

Research combine disease phenotype similarity, semantic similarity, and miRNA-disease network to calculate miRNAs functional similarity [30, 31].

For the two miRNAs *m*_*i*_ and *m*_*j*_, (1) According to the miRNA-disease network, we set *MD*_*i*_ = {*md*_*1*_, *md*_*2*_, …, *md*_*ni*_} for all the diseases associated with *m*_*i*_, and *MD*_*j*_ = {*md*_*1*_, *md*_*2*_, …, *md*_*nj*_} for all the diseases associated with *m*_*j*_. (2) We calculate the semantic value of each disease in *MD*_*i*_ and *MD*_*j*_. (3) Finally, the functional similarity of *m*_*i*_ and *m*_*j*_ is calculated as follows:

7 $$FSM\left( {m_{i} ,m_{j} } \right) = \frac{{\sum\nolimits_{{1 \le p \le n_{j} }} {S\left( {md_{p} ,MD_{i} } \right) + } \sum\nolimits_{{1 \le q \le n_{i} }} {S\left( {md_{q} ,MD_{j} } \right)} }}{{n_{i} + n_{j} }}$$

Respectively, *n*_*i*_ is the number of diseases associated with *m*_*i*_. *n*_*j*_ is the number of diseases associated with *m*_*j*_. *S(md, MD) i*s the max semantic similarity between the disease *md* and any diseases in another set *MD.*

### Disease and miRNA similarity

As mentioned above, the Gaussian interaction kernel function is used for computing the disease and miRNA similarity [32].

In the miRNA-disease association network, the binary interaction profile vector *IP(x*_*i*_*)* represents the interaction information of disease or miRNA. Therefore, the Gaussian interaction profile kernel similarity for diseases or miRNAs is defined as follows:

8 $$GS_{x} \left( {x_{i} ,x_{j} } \right) = \exp \left( { - \gamma _{x} \left\| {IP(x_{i} ) - IP(x_{j} )} \right\|^{2} } \right)$$

In the formula, *x* can represent disease *d* or miRNA *m*, *IP(x*_*i*_*)* is the interaction information of disease *d*_*i*_ or miRNA *m*_*i*_. *IP(x*_*j*_*)* is the interaction information of disease *d*_*j*_ or miRNA *m*_*j*_.

*γ*_*x*_ is a parameter controlling the kernel bandwidth and can be calculated by normalizing *γ*_*x*_*’* by the average number of related miRNAs(diseases) per disease(miRNA). The specific formula is as follows:

9 $$\gamma _{x} = \gamma _{x}^{\prime } /\left( {\frac{1}{{n_{x} }}\sum\limits_{{i = 1}}^{{n_{x} }} {\left\| {IP(x_{i} )} \right\|} ^{2} } \right)$$

Here, we set *γ*_*x*_*’* to a value of 1 based on the previous study [33], so that we can have a better comparison.

### Integrated similarity for diseases and miRNAs

To deal with the problem that some diseases have no semantic similarity or miRNAs have no functional similarity, here we propose a reasonable method: if *SS(d*_*i*_, *d*_*j*_*)* (the semantic similarity of disease *d*_*i*_ and *d*_*j*_) exists, the similarity of these two diseases will finally be

10 $$\frac{{GS_{d} \left( {d_{i} ,d_{j} } \right) + SS\left( {d_{i} ,d_{j} } \right)}}{2}$$

the average of Gaussian interaction profile kernel similarity and semantic similarity; otherwise, it will be only *GS*_*d*_*(d*_*i*_, *d*_*j*_*)* (Gaussian interaction profile kernel similarity). In the same way, if *FSM(m*_*i*_, *m*_*j*_*)* (the functional similarity of miRNA *m*_*i*_ and *m*_*j*_) exists, the similarity of these two miRNAs will finally be

11 $$\frac{{GS_{m} \left( {m_{i} ,m_{j} } \right) + FSM\left( {m_{i} ,m_{j} } \right)}}{2}$$

the average of Gaussian interaction profile kernel similarity and functional similarity; otherwise, it will be only *GS*_*m*_*(m*_*i*_, *m*_*j*_*)* (Gaussian interaction profile kernel similarity).

### Noise visualization

From HMDD v2.0, we download 5430 miRNA-disease associations as a positive sample. According to the research in AEMDA [24], there are 12,034 known pairs in HMDD v3.0. Therefore, if we choose negative samples randomly, we estimate that it will obtain the data containing about 3.59% of the noise.

**Fig. 6:**
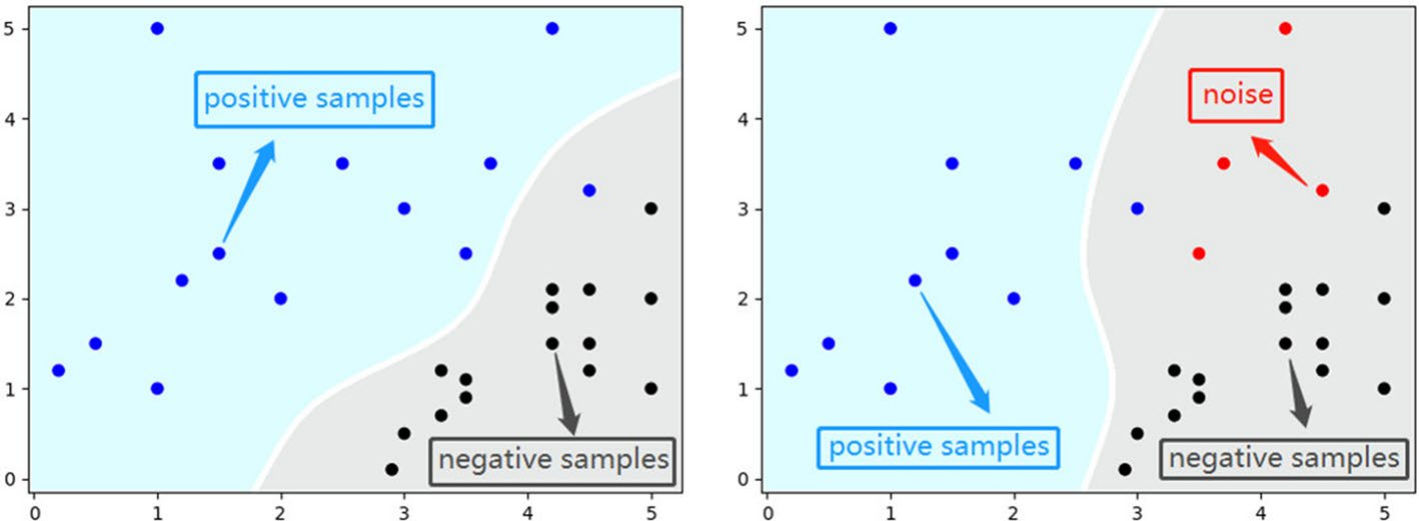
The interference of the noise hiding in the dataset

To illustrate the impact of the noise, we design the experiment as follows:

1. First, we extract 200 positive samples and 200 negative samples as noise-free data from the UCI ML Breast Cancer Wisconsin (Diagnostic) dataset [34].
2. Then, we deliberately change 7 positive samples’ labels in the noise-free data into negative labels to simulate the noise hiding in data and form the noise data. The situation process is shown in Fig. 6. The red dots represent the noise hiding in the data. The blue dots and the black ones represent positive samples and negative samples, respectively. It is shown that the decision boundaries are different because of the noise in the two situations.
3. Further, we maintain positive samples and negative samples 200 each in the noisy data to make sure the experiment is rigorous.
4. Finally, we use the logistic regression algorithm on both noise-free and noise data to demonstrate the interference caused by the noise. The results are listed in Table 3.

**Table 3:** The performance of logistic regression algorithm on noise and noise-free data

Data	AUROC	AUPR	Precision	Recall	F1-score
Noise-free	0.9897	0.9892	0.9273	0.9450	0.9344
Noise	0.9647	0.9519	0.9039	0.9400	0.9204

Further, the experiments can prove that the noise hiding in the data affects the final results of miRNA-disease associations prediction to a certain extent. To be specific, the noise hiding in the data is close to positive samples, which can cause interference to algorithms on judging positive samples.

### Method for negative samples selection

Inspired by ABMDA [19], here we use the *k*-means algorithm [35] to select negative samples. The specific process is as follows: we cluster all undetected miRNA-disease pairs into 23 clusters by *k*-means. The similar pairs will be in the same cluster after clustering, which makes the noise in the same cluster and distinguished easily. Then, we extract equal amounts of samples from each cluster as negative samples in a way that the noise can be reduced to some extent.

### Anti-noise computational model for miRNA-disease associations prediction

To further resist the noise, we propose a subsampling method for noise smoothing motivated by Ho [36]. In detail, we construct several subsets by sampling with replacement from the original data.

Then, we feed each subset to LightGBM [37], which is an ensemble algorithm based on GBDT [38]. In each learning iteration, the basic model of LightGBM learns the residual result from the previous iteration so that it can improve the performance. What’s more, LightGBM utilizes two significant techniques: Gradient-based One-Side Sampling (GOSS) for data samples and Exclusive Feature Bundling (EFB) for features. To be specific, GOSS can maintain the examples with large gradients and randomly picks examples with small gradients, which reduces the training cost. EFB can bundle many exclusive features to fewer dense features, which further reduces the cost of calculating for zero feature values.

**Fig. 7:**
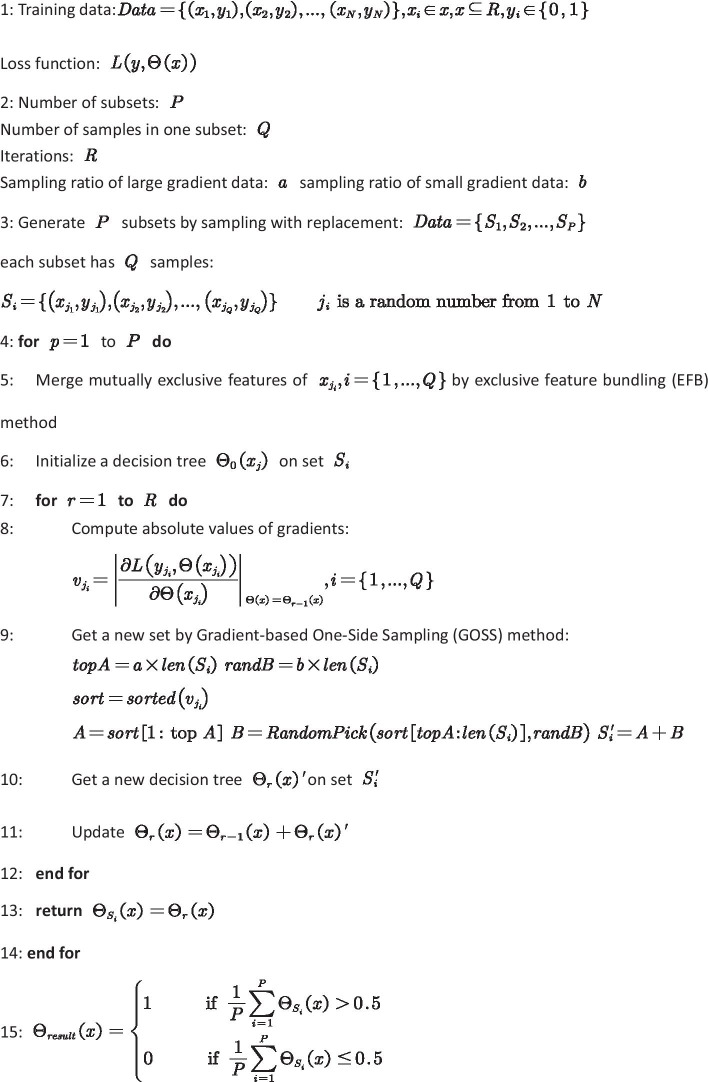
The pseudocode of ANMDA

The eventual result is an average of each subset’s prediction result. The detailed steps of the ANMDA are shown in Fig. 7.

## Supplementary Information

**Additional file 1**. The top 200 miRNA-disease associations predicted by ANMDA

**Additional file 2**. The case studies of ANMDA

## Data Availability

The data and materials are available from https://github.com/BioInfoLeo/ANMDA

## Abbreviations

miRNA: MicroRNA
ANMDA: Anti-noise algorithm for predicting miRNA-disease associations
LightGBM: Light gradient boosting machine
HMDD: Human microRNA disease database
ncRNA: Non-coding RNA
ROC: Receiver operating characteristic
PR: Precise-recall
AUROC: Area under the receiver operating characteristic curve
AUPR: Area under the precise-recall curve
kNN: *k*-Nearest neighbor
MLP: Multilayer perceptron
DAG: Directed acyclic graph
GOSS: Gradient-based one-side sampling
EFB: Exclusive feature bundling
