# Supplementary material for: ANMDA: anti-noise based computational model for predicting potential miRNA-disease associations
Source: BMC Bioinformatics. 2021 Jul 2;22:358. doi: 10.1186/s12859-021-04266-6 (PMC8254275; doi:10.1186/s12859-021-04266-6)
Supplement: Supplementary file 2 — Additional file 2. The case studies of ANMDA [file 12859_2021_4266_MOESM2_ESM.doc]

*Table 1: The top 50 miRNA-disease associations predicted by ANMDA*

| **Disease** | **miRNA** | **Evidence (pmid)** |
| --- | --- | --- |
| Gastric Neoplasms | hsa-let-7d | 19156147 |
| Gastric Neoplasms | hsa-let-7c | 19156147 |
| Colorectal Neoplasms | hsa-let-7d | 17965831 |
| Gastric Neoplasms | hsa-mir-29b | 25874772 |
| Gastric Neoplasms | hsa-let-7b | 19156147 |
| Gastric Neoplasms | hsa-let-7e | 19156147 |
| Carcinoma, Hepatocellular | hsa-mir-133a | 26173501 |
| Gastric Neoplasms | hsa-mir-19b | 29872807 |
| Lung Neoplasms | hsa-mir-106b | 27797825 |
| Gastric Neoplasms | hsa-mir-210 | 26059512 |
| Gastric Neoplasms | hsa-mir-133a | 25620172 |
| Colorectal Neoplasms | hsa-mir-29b | 29545333 |
| Colorectal Neoplasms | hsa-mir-132 | 29017096 |
| Colorectal Neoplasms | hsa-mir-106b | 21283757 |
| Colorectal Neoplasms | hsa-mir-29c | 28392396 |
| Colorectal Neoplasms | hsa-let-7f | 25330373 |
| Carcinoma, Hepatocellular | hsa-mir-132 | 30377095 |
| Gastric Neoplasms | hsa-mir-15a | 26894855 |
| Colorectal Neoplasms | hsa-mir-223 | 29152124 |
| Melanoma | hsa-mir-24 | unconfirmed |
| Colorectal Neoplasms | hsa-mir-15a | 22574716 |
| Carcinoma, Hepatocellular | hsa-mir-429 | 25931210 |
| Colorectal Neoplasms | hsa-mir-24 | 27888625 |
| Gastric Neoplasms | hsa-mir-205 | 27082508 |
| Carcinoma, Hepatocellular | hsa-mir-34b | 24704024 |
| Melanoma | hsa-mir-29c | unconfirmed |
| Gastric Neoplasms | hsa-mir-203 | 25373785 |
| Colorectal Neoplasms | hsa-mir-30b | 29152124 |
| Gastric Neoplasms | hsa-mir-92a | 23868977 |
| Melanoma | hsa-mir-92a | 27620505 |
| Colorectal Neoplasms | hsa-mir-214 | 27537384 |
| Melanoma | hsa-mir-223 | 23111773 |
| Carcinoma, Hepatocellular | hsa-mir-143 | 25270212 |
| Carcinoma, Squamous Cell | hsa-let-7a | 27835588 |
| Gastric Neoplasms | hsa-let-7i | 19156147 |
| Carcinoma, Hepatocellular | hsa-mir-206 | 25391771 |
| Colorectal Neoplasms | hsa-mir-205 | 20859756 |
| Colorectal Neoplasms | hsa-mir-192 | 19074875 |
| Carcinoma, Squamous Cell | hsa-mir-17 | unconfirmed |
| Lung Neoplasms | hsa-mir-195 | 28752530 |
| Lung Neoplasms | hsa-mir-141 | 25910758 |
| Melanoma | hsa-mir-150 | 30132912 |
| Carcinoma, Hepatocellular | hsa-mir-9 | 29291025 |
| Colorectal Neoplasms | hsa-mir-30c | 29152124 |
| Colonic Neoplasms | hsa-mir-210 | 29152124 |
| Melanoma | hsa-mir-10b | 26208390 |
| Melanoma | hsa-mir-132 | 28767374 |
| Prostatic Neoplasms | hsa-mir-210 | 29901117 |
| Gastric Neoplasms | hsa-mir-26a | 27010210 |
| Carcinoma, Hepatocellular | hsa-mir-23b | 29901200 |

*Table 2: The top 10 miRNAs associated with different diseases predicted by ANMDA.*

*(a): The top 10 miRNAs associated with prostate neoplasm*

| **Disease** | **miRNA** | **Evidence (pmid)** |
| --- | --- | --- |
| Prostatic Neoplasms | hsa-mir-210 | 29901117 |
|  | hsa-mir-24 | 29246734 |
|  | hsa-mir-30b | unconfirmed |
|  | hsa-mir-150 | 29441850 |
|  | hsa-mir-29c | 29715514 |
|  | hsa-mir-429 | 20539944 |
|  | hsa-let-7f | unconfirmed |
|  | hsa-mir-206 | 29805562 |
|  | hsa-mir-7 | 24760272 |

*(b): The top 10 miRNAs associated with gastric neoplasm*

| **Disease** | **miRNA** | **Evidence (pmid)** |
| --- | --- | --- |
| Gastric Neoplasms | hsa-let-7d | 19156147 |
|  | hsa-let-7c | 19156147 |
|  | hsa-mir-29b | 25874772 |
|  | hsa-let-7b | 19156147 |
|  | hsa-let-7e | 19156147 |
|  | hsa-mir-19b | 29872807 |
|  | hsa-mir-210 | 26059512 |
|  | hsa-mir-133a | 25620172 |
|  | hsa-mir-15a | 26894855 |
|  | hsa-mir-205 | 27082508 |

*(c): The top 10 miRNAs associated with colorectal carcinoma*

| **Disease** | **miRNA** | **Evidence (pmid)** |
| --- | --- | --- |
| Colorectal Neoplasms | hsa-let-7d | 17965831 |
|  | hsa-mir-29b | 29545333 |
|  | hsa-mir-132 | 29017096 |
|  | hsa-mir-106b | 21283757 |
|  | hsa-mir-29c | 28392396 |
|  | hsa-let-7f | 25330373 |
|  | hsa-mir-223 | 29152124 |
|  | hsa-mir-15a | 22574716 |
|  | hsa-mir-24 | 27888625 |
|  | hsa-mir-30b | 29152124 |

*(d): The top 10 miRNAs associated with melanoma*

| **Disease** | **miRNA** | **Evidence (pmid)** |
| --- | --- | --- |
| Melanoma | hsa-mir-24 | unconfirmed |
|  | hsa-mir-29c | unconfirmed |
|  | hsa-mir-92a | 27620505 |
|  | hsa-mir-223 | 23111773 |
|  | hsa-mir-150 | 30132912 |
|  | hsa-mir-10b | 26208390 |
|  | hsa-mir-132 | 28767374 |
|  | hsa-mir-206 | 21763111 |
|  | hsa-mir-30c | unconfirmed |
|  | hsa-mir-101 | 23962556 |

*(e): The top 10 miRNAs associated with hepatocellular carcinoma*

| **Disease** | **miRNA** | **Evidence (pmid)** |
| --- | --- | --- |
| Hepatocellular Carcinoma | hsa-mir-133a | 26173501 |
|  | hsa-mir-132 | 30377095 |
|  | hsa-mir-429 | 25931210 |
|  | hsa-mir-34b | 24704024 |
|  | hsa-mir-143 | 25270212 |
|  | hsa-mir-206 | 25391771 |
|  | hsa-mir-9 | 29291025 |
|  | hsa-mir-23b | 29901200 |
|  | hsa-mir-27b | 27704356 |
|  | hsa-mir-204 | 23282077 |
